# Changes in Olfactory Bulb Volume in Parkinson’s Disease: A Systematic Review and Meta-Analysis

**DOI:** 10.1371/journal.pone.0149286

**Published:** 2016-02-22

**Authors:** Jia Li, Cheng-zhi Gu, Jian-bin Su, Lian-hai Zhu, Yong Zhou, Huai-yu Huang, Chun-feng Liu

**Affiliations:** 1 Department of Neurology, Second Affiliated Hospital of Soochow University, Suzhou, 215004, China; 2 Institute of Neuroscience, Soochow University, Suzhou 215123, China; 3 Department of Neurology, Second Affiliated Hospital of Nantong University, Nantong, 226001, China; 4 Department of Endocrinology, Second Affiliated Hospital of Nantong University, Nantong, 226001, China; University of New Mexico, UNITED STATES

## Abstract

**Objective:**

The changes in olfactory bulb (OB) volume in Parkinson’s disease (PD) patients have not yet been comprehensively evaluated. The purpose of this meta-analysis was to explore whether the OB volume was significantly different between PD patients and healthy controls.

**Methods:**

PubMed and Embase were searched up to March 6, 2015 with no language restrictions. Two independent reviewers screened eligible studies and extracted data on study characteristics and OB volume. Additionally, a systematic review and meta-analysis using a random-effects model were conducted. Publication bias was determined by using funnel plots and Begg’s and Egger’s tests. Subgroup analyses were performed to assess possible sources of heterogeneity.

**Results:**

Six original case-control studies of 216 PD patients and 175 healthy controls were analyzed. The pooled weighted mean difference (WMD) in the OB volume between the PD patients and the healthy participants was -8.071 for the right OB and -10.124 for the left OB; these values indicated a significant difference among PD patients compared with healthy controls. In addition, a significant difference in the lateralized OB volume was observed in PD patients, with a pooled WMD of 1.618; these results indicated a larger right OB volume than left OB volume in PD patients. In contrast, no difference in the lateralized OB volume was found in healthy controls. No statistical evidence of publication bias among studies was found based on Egger’s or Begg’s tests. Sensitivity analyses revealed that the results were consistent and robust.

**Conclusions:**

Overall, both the left and the right OB volume were significantly smaller in PD patients than in healthy controls. However, significant heterogeneity and an insufficient number of studies underscore the need for further observational research.

## Introduction

Parkinson’s disease (PD) is the most common progressive neurological disorder that severely threatens human health and quality of life. The incidence of PD is expected to rise steadily in the future as the human population ages [1–3]. Olfactory dysfunction is one of the most common non-motor symptoms of PD, and its prevalence is nearly 90% [4,5]. Moreover, olfactory dysfunction an early symptom of PD that often precedes motor symptoms by several months or even years [6]. A recent autopsy study by Hawkes revealed significant neuronal loss in the anterior olfactory nucleus in PD patients, and this finding suggested a strong association between changes in the olfactory bulb (OB) and PD-associated olfactory dysfunction [7]. These olfactory deficiencies have been associated to functional and/or structural changes at the level of the OB [8–10], which is an important component of the olfactory system. The volume of the OB can reliably be evaluated via magnetic resonance imaging (MRI) in vivo. Analyzing the OB volume has been suggested to assist in early diagnosis and differential of PD [10–13]. However, a series of studies on the changes in OB volume observed between PD patients and healthy controls have reported variable findings. To resolve this controversial issue, a systematic review and meta-analysis was performed to comprehensively assess whether the OB volume was significantly different between PD patients and healthy controls.

## Materials and Methods

This meta-analysis was performed according to the Preferred Reporting Items for Systematic Reviews and Meta-Analyses (PRISMA) statement (**S1 Checklist**) [14].

### Search strategy

A systematic and comprehensive search was conducted up to March 6, 2015, with no language restrictions using the following electronic databases: 1) PubMed (http://www.ncbi.nlm.nih.gov/pubmed) and 2) Embase (www.embase.com). The literature search included Medical Subject Headings (MeSH) terms, terms from the Emtree thesaurus and related free-text terms in a manner that combined ‘Parkinson Disease’ and ‘Olfactory Bulb Volume’. Detailed search strategies for these two databases are presented in **S1 Appendix**. In addition, the reference lists from all included studies were examined.

### Study selection and inclusion criteria

Retrieved studies from both PubMed and Embase were imported into Endnote (version X6; Thomson Reuters), where duplications were deleted manually. Two independent reviewers (i.e., JL and JBS) screened the titles and the abstracts of the remaining studies. In addition, the full-text version was retrieved to further evaluate the study’s topic if that information could not easily be determined based on its title or abstract. Disagreements between reviewers were settled by discussion or by consultation with a third reviewer (i.e., CZG). Conference abstracts were excluded in the analysis because of the uncertainty of the study quality and concerns with regard to the reporting of insufficient data.

Studies were eligible for inclusion if they met the following criteria: (1) original study was a case-control study; (2) patients with PD in the study were compared with healthy controls who were at least matched according to age, if possible; (3) the study established specific clinical criteria for the diagnosis of PD; (4) the OB volume was evaluated via analysis of an MRI scan; and (5) the OB volume was expressed as the means and standard deviation (SD) in both groups, or the means and SD could be calculated from the data for these variables. Letters, commentaries, review articles and case reports were excluded. If possible, the authors of the included studies were contacted in cases of unpublished or insufficient data.

### Data extraction

Two reviewers (i.e., JL and LHZ) independently selected the records and extracted the data from the eligible studies. A standardized data extraction form was used, and baseline characteristics including publication year, study country, PD diagnosis criteria, sample size, gender, mean age, Unified Parkinson's Disease Rating Scale of motion (i.e., the UPDRS-III scale) scores in PD patients, the Hoehn and Yahr scale (H-Y scale) scores, scores on a cognitive function assessment scale (Mini-Mental State Examination (MMSE) or Moca scale), olfactory test results, magnetic field intensity of MRI, and the volume of both the left and the right OB were obtained. Disagreements were settled by consultation with a third party (i.e., YZ) until both reviewers reached an agreement or by contacting relevant experts if necessary.

### Study quality assessment

Each study that was included was assessed independently by two reviewers (i.e., JL and LHZ) using an established form for case-control studies, the Newcastle-Ottawa Scale (NOS) [15], which is used to assess the quality of observational studies. Discrepancies were reported to and settled by a third party (i.e., HYH). Three major aspects of study quality were scored: 1) selection of the study groups (0–4 points); 2) determination of the exposure of interest in the studies (0–3 points); and 3) quality of the adjustment for confounding variables (0–2 points). A study could be scored a maximum of one star for each item numbered within the categories of Selection and Exposure, while at most two stars could be allocated to Comparability. A higher score represented improved greater quality of the study methodology. A score equal to or higher than six was considered indicate high study quality.

### Statistical analysis

For continuous variables, means and SDs were used to calculate the weighted mean difference (WMD). Data expressed as means and SDs were obtained from available studies, and data that were not directly expressed in this manner were calculated using the sample size and standard error (SE). The statistical software package Stata (version 12.0; Stata Corporation, College Station, TX, USA) was used to perform the quantitative synthesis. Summary estimates, including 95% confidence intervals (CIs), were calculated. The Cochrane *Q* test and the Higgins *I*^*2*^ index were used to assess the heterogeneity among studies [16]. A fixed-effects model was adopted for the analysis if the *P* value of the *Q* statistic was > 0.1 and if the *I*^*2*^ index was < 50%, as these results would indicate no between-study heterogeneity. Otherwise, a random-effects model was applied [17]. Sensitivity analyses were applied to evaluate the influence of each individual study on the stability of the overall pooled estimate. Subgroup analyses were applied according to the country of origin of the participants and the magnetic field intensity of MRI to explore the inherent heterogeneity in the primary analyses. Publication bias was evaluated visually using funnel plots and statistically using Begg’s and Egger’s regression models [18], in which a P-value <0.10 was considered statistically significant [19].

## Results

### Literature search

**Fig 1:**
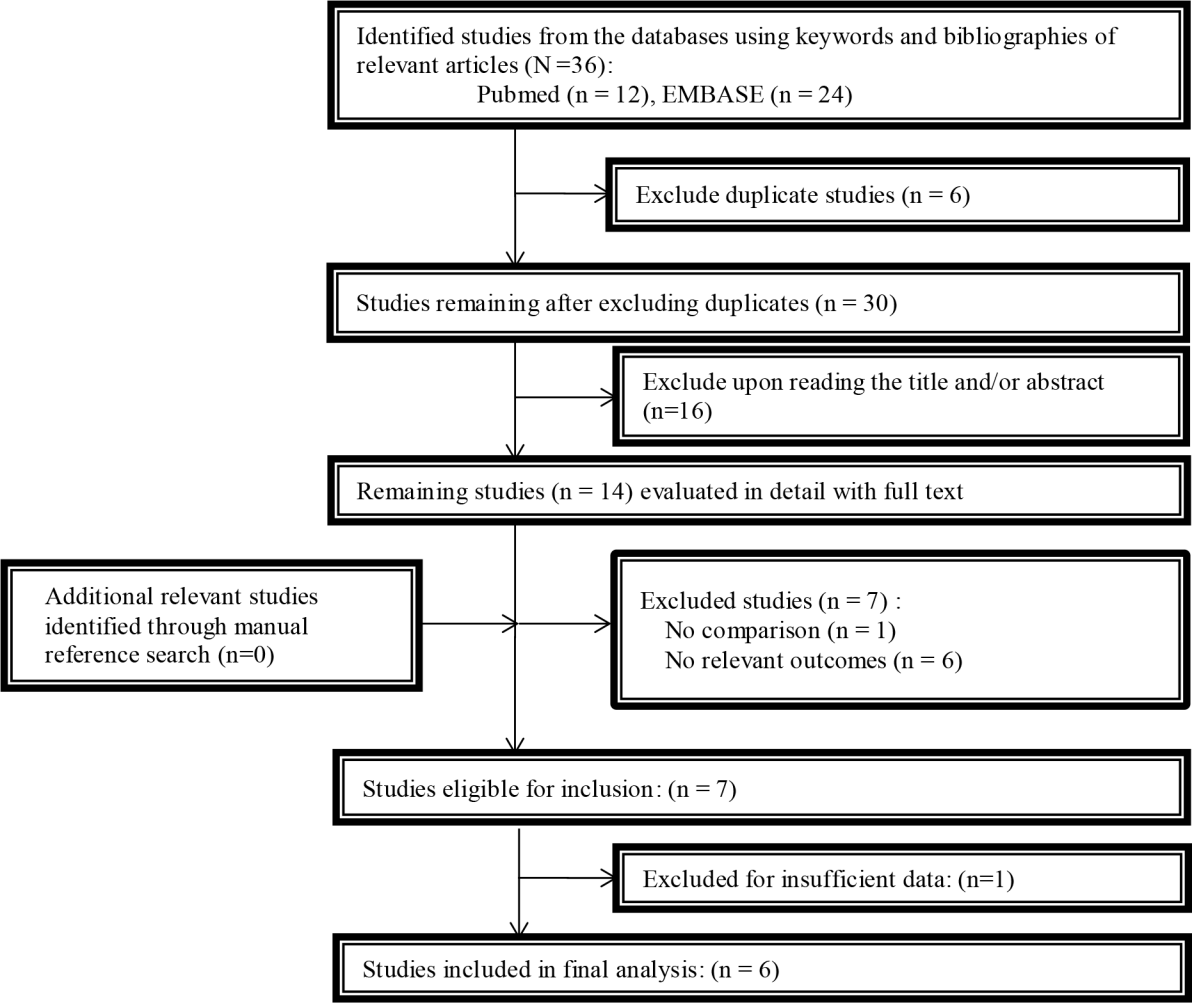
Flow chart of the study selection process.

Fig 1 presents the study selection process. Briefly, a total of 36 publications were identified from the initial search. Next, six records were excluded after duplication screening, and 16 records were excluded based on title and abstract evaluation. Thus, 14 potentially relevant records were further reviewed by full text reading [13,20–32]. However, seven of these records were conference abstracts, which were excluded due to uncertainty concerning study quality and data sufficiency, as no detailed diagnostic criteria or characteristics of comparison and exposure were found, and the data for the OB volume was found to be insufficient [23–26,29–31]. Of the remaining seven records, the OB volume data were expressed as the means and SDs in four studies [20,21,27,28]. The corresponding authors of another three studies were contacted via e-mail to resolve unclear and unpublished data. The author of only one study in which the OB volume was demonstrated in graphic form sent us the original data of his research [13], and the two other authors failed to respond. Thus, based on the SE and the sample size in one study, the SD was calculated for the meta-analysis [22]. However, due to insufficient data in which the OB volume was expressed as the mean and SE and was reported as the “best” volume (meaning the larger of the left or the right OB volume) rather than both the left and the right OB volume, this study was excluded from quantitative analysis [32]. Ultimately six studies were included in the analysis.

### Study characteristics and quality assessment results

The baseline characteristics and the quality assessment of each study are summarized in Table 1. The study design variants and the overall results of subgroup analyses are presented in Tables 2 and 3, respectively.

**Table 1:** Baseline Characteristics and Quality Assessment of Included Studies.

Baseline Characteristics and Quality Assessment of Included Studies															
First author	Year	Country	Diagnostic criteria	Sample size(female)		Adjustment variables	Motor Function	Magnetic field intensity(Tesla)	Olfactory test	Cognitive function	Blind method	Newcastle-Ottawa Scale (NOS)			
				PD	HC							Selection	Comparability	Exposure	Total score
Altinayar,S	###	Turkey	UKBB	41(17)	19(9)	age,gender	UPDRS-Ⅲ, H-Y scale	1.5T	CCCRC	Not mentioned	Yes	3	2	2	7
Brodoeh,S	###	Germany	clinical	16(8)	16(8)	age,gender	UPDRS-Ⅲ, H-Y scale	3.0T	Sniffin' Sticks	MMSE	Yes	4	2	2	8
Chen,S	###	China	UKBB	20(9)	12(5)	age,MMSE	UPDRS-Ⅲ, H-Y scale	1.5T	Not mentioned	MMSE,Moca	Yes	3	2	2	7
Hakyemez, H. A.	###	Turkey	clinical	28(5)	19(8)	age	UPDRS-Ⅲ	1.5T	UPSIT	Not mentioned	Yes	3	2	2	7
Hang,W	###	China	clinical	100(47)	100(49)	age,gender, MMSE,education	UPDRS-Ⅲ, H-Y scale	3.0T	T&T	MMSE,Moca	Not mentioned	3	2	2	7
Muller,A	###	Germany	clinical	11(1)	9(6)	age	UPDRS-Ⅲ	1.5T	Sniffin' Sticks	Not mentioned	Not mentioned	3	2	1	6

Abbreviations: UKBB = United Kingdom PD Society Brain Bank diagnostic criteria;UPDRS-Ⅲ = Unified Parkinson's Disease Rating Scale; CCCRC = Connecticut Chemosensory Clinical Research Center; MMSE = Mini-Mental State Examination;UPSIT = University of Pennsylvania Smell Identification Test.

**Table 2:** OB volume in PD patients and healthy controls.

OB volume in PD patients and healthy controls									
First author	Year	Magnetic field intensity(Tesla)	country	Sample size(female)		Right OB volume(mean±SD) (mm^3^)		Left OB volume(mean±SD)(mm^3^)	
				PD	HC	PD	HC	PD	HC
Altinayar,S	2014	1.5T	Turkey	41(17)	19(9)	40.06±13.45	44.05±19.23	38.89±13.71	41.84±14.76
Brodoeh,S	2012	3.0T	Germany	16(8)	16(8)	46.40±11.48	66.20±12.95	41.50±10.06	65.2±13.01
Chen,S	2013	1.5T	China	20(9)	12(5)	27.80±16.20	37.80±11.40	27.30±16.80	38.10±8.50
Hakyemez,H	2012	1.5T	Turkey	28(5)	19(8)	74.50±20.24	68.26±12.47	72.25±22.58	70.05±15.82
Hang,W	2015	3.0T	China	100(47)	100(49)	35.79±5.28	47.75±6.51	34.25±5.14	47.38±6.47
Muller,A	2005	1.5T	Germany	11(1)	9(6)	54.50±26.94	57.73±24.96	54.05±21.38	60.60±27.17

Abbreviations: PD = Parkinson's disease; HC = healthy controls.

**Table 3:** Subgroup analyses of OB volume in PD patients and healthy controls.

Subgroup analyses of OB volume in PD patients and healthy controls						
			Overall effect		Heterogeneity	
Subgroup characteristic	No. of studies	OB volume side	Summery WMD	95% CI of WMD	*I*^*2*^ test(%)	P-value
***Region of origin***						
Germany	2	R	-15.03	-29.734, -0.325	43.90%	0.182
		L	-18.235	-33.897, -2.573	52.20%	0.148
China	2	R	-11.904	-13.523, -10.285	0.00%	0.693
		L	-13.054	-14.646, -11.461	0.00%	0.61
Turkey	2	R	1.177	-8.848, 11.202	55.40%	0.134
		L	-1.207	-7.594, 5.180	0.00%	0.455
***Magnetic field intensity of MRI***						
1.5T	4	R	-2.59	-10.430, 5.251	48.20%	0.122
		L	-4.566	-10.234, 1.103	16.30%	0.31
3.0T	2	R	-14.731	-22.077, -7.385	68.40%	0.075
		L	-17.648	-27.896, -7.399	84.30%	0.012

Abbreviations: WMD = weighted mean difference; R = right; L = left.

The included studies were published between 2005 and 2015. The total sample size of all studies was 391 (i.e., 216 PD patients and 175 healthy controls), with an average of 65 participants per study and a range from 11 to 100 participants per study. The UPDRS-III scale was evaluated in all studies [13,20–22,27,28]. Two studies were performed in Turkey [20,27], two were conducted in Germany [13,21], and the remaining two studies were applied in China [22,28]. Olfactory function was tested using the Sniffin’ Sticks battery in two studies [13,21], T&T olfactometry in one study [28], the Connecticut Chemosensory Clinical Research Center (CCCRC) test in one study [20], and the University of Pennsylvania Smell Identification Test (UPSIT) was performed in one study [27]. All of these tests indicated the loss of olfaction in PD patients compared with healthy controls, although this result was not mentioned in one study [22]. The MMSE was used for cognitive assessment in three studies [13,21,22], but the MMSE was not mentioned in the other three reports [20,27,28]. The OB volume was evaluated using a 1.5T MRI imaging system in four studies [13,20,22,27] and a 3.0T MRI imaging system in the remaining two studies [21,28]. The observer of the OB volume was blinded to the diagnosis in four studies [20–22,27], but blinding was not mentioned in two studies [13,28]. Of all six included studies, three reported that the OB volume was significantly decreased in PD patients compared with healthy controls [21,22,28], whereas two found no significant difference [13,20] and one reported a higher OB volume in PD patients than in controls [27].

Quality assessment of the individual studies showed that the NOS score ranged from six to eight. These results indicated that the quality of methodology was generally good.

### Right OB volume between PD patients and healthy controls

**Fig 2:**
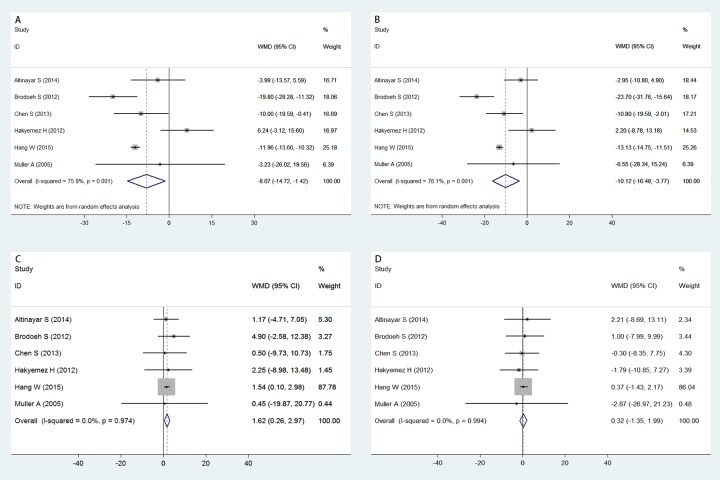
Forest plots of the meta-analysis for OB volume differences. PD patients vs healthy controls for right OB volume (A); PD patients vs healthy controls for left OV volume (B); OB volume in PD patients (C); OB volume in healthy controls (D). The size of the square surrounding each effect estimate indicates the weight of the individual study in the meta-analysis, and the horizontal bars indicate the corresponding 95% CIs. The diamond represents the pooled WMD, and the line width represents the 95% CIs. WMD = weighted mean difference; CI = confidence interval.

**Fig 3:**
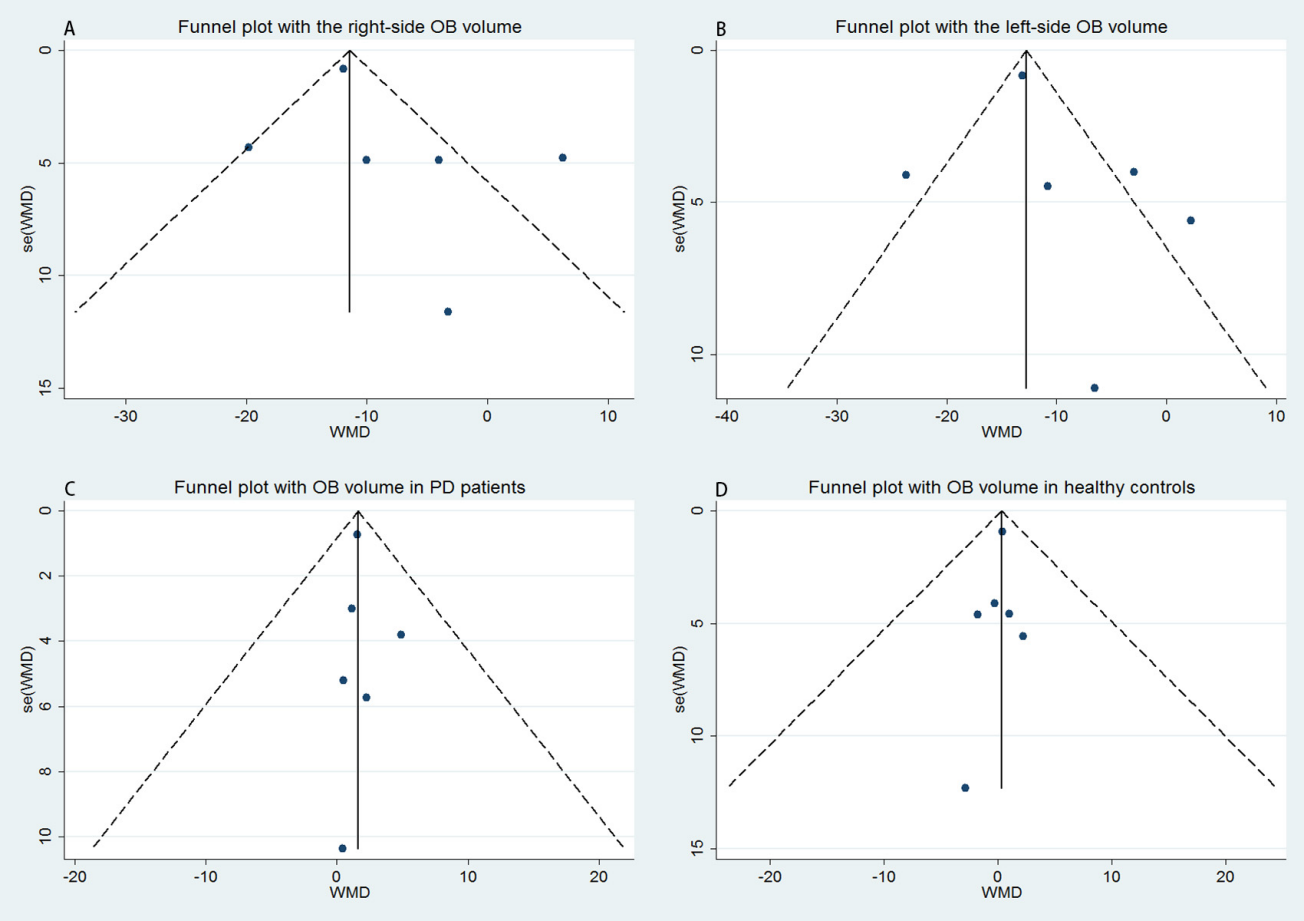
Funnel plots of the relationship between the size of the effect of individual studies and the precision of the study estimate (WMD, horizontal axis; s.e., vertical axis) for the right OB volume (A), the left OB volume (B), and the OB volume in PD patients (C) and in healthy controls (D). WMD = weighted mean difference.

**Fig 4:**
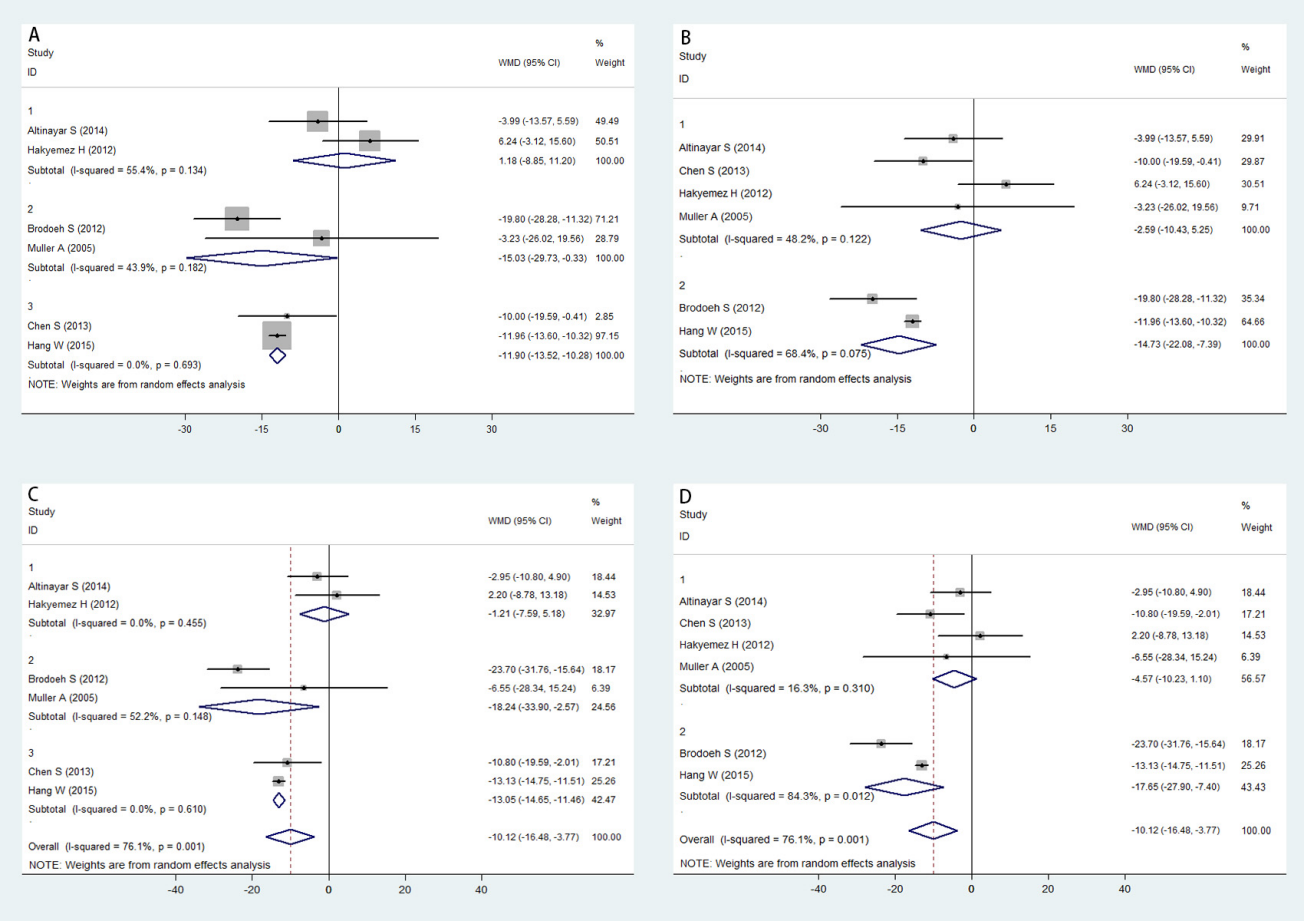
Subgroup analyses with forest plots of the following comparisons: effect of country of origin on the right OB volume (A), effect of magnetic intensity of MRI on the right OB volume (B), effect of country of origin on the left OB volume (C), and effect of magnetic intensity of MRI on the left OB volume (D). The squares represent the WMD estimates for each individual study, and the area of the squares reflects the weight assigned to the studies. The horizontal line across each square represents the corresponding 95% CIs. The diamond represents the summary WMD, and the line width represents the 95% CIs. WMD = weighted mean difference; CI = confidence interval.

Six studies were pooled for comparison of the right OB volume between PD patients and healthy controls. All studies indicated that the right OB volume in PD patients was significantly smaller than that in healthy controls (WMD = -8.071; 95% CI -14.721, -1.421; Fig 2A). The funnel plot for the right OB volume based on studies of PD patients and healthy controls was examined visually, and the contour of the funnel plot appeared symmetrical (Fig 3A). No statistical evidence of any publication bias among the analyzed studies based on either Begg’s test (P = 0.707) or Egger’s test (P = 0.372) was found. However, the random-effects model was used because the *I*^*2*^ index was 75.9% and the *Q* statistic considering five degrees of freedom was 20.73 (P = 0.001), indicating substantial heterogeneity. Therefore, subgroup analyses were conducted to evaluate the effect of between-study variability on the pooled results according to the country of origin of the participants and the magnetic field intensity of MRI. Alternatively, sensitivity analyses were applied to evaluate the influence of each study on the stability of the overall pooled estimate. After stratification by country of origin, the differences in right OB volume between PD patients and healthy controls were similar between studies of German (WMD = -15.030; 95% CI -29.734, -0.325; P_*Q*_ = 0.182; *I*^*2*^ = 43.9%) and Chinese populations (WMD = -11.904; 95% CI -13.523, -10.285; P_*Q*_ = 0.693; *I*^*2*^ = 0), but these results were different from those in studies of Turkish populations (WMD = 1.177; 95% CI -8.848, 11.202; P_*Q*_ = 0.134; *I* ^*2*^ = 55.4%; Fig 4A). Upon stratification by the magnetic field intensity of MRI, the OB volume results were similar between studies performed 3.0T MRI (WMD = -14.731; 95% CI -22.077, -7.385; P_*Q*_ = 0.075; *I*^*2*^ = 68.4%); however, these results were distinct from those of the studies that performed 1.5T MRI (WMD = -2.590; 95% CI -10.430, 5.251; P_*Q*_ = 0.122; *I*^*2*^ = 48.2%; Fig 4B). Sensitivity analyses showed no significant changes in the pooled WMD or 95% CI upon excluding any of the studies. This finding suggested that the overall pooled estimates were stable and robust.

### Left OB volume between PD patients and healthy controls

The left OB volume was significantly smaller in PD patients than in healthy controls according to the pooled analysis of six studies (WMD = -10.124; 95% CI -16.476, -3.773; Fig 2B). The results of Begg’s test (P = 0.452), Egger’s test (P = 0.478), and funnel plots provided no evidence of publication bias (Fig 3B). A random-effects model was used because significant heterogeneity was detected (P_*Q*_ = 0.001; *I*^*2*^ = 76.1%). Subgroup analyses were applied according to the country of origin of the participants and the magnetic field of intensity of MRI, and sensitivity analyses were conducted to evaluate the stability of the overall pooled estimates. Upon stratification by country of origin, the left OB volume was similar between studies of German (WMD = -18.235;95% CI -33.897, -2.573; P_*Q*_ = 0.148; *I*^*2*^ = 52.2%) and Chinese populations (WMD = -13.054; 95% CI -14.646, -11.461; P_*Q*_ = 0.610; *I*^*2*^ = 0), but these results were different from those of studies of Turkish populations (WMD = -1.207; 95% CI -7.594, 5.180; P_*Q*_ = 0.455; *I* ^*2*^ = 0%; Fig 4C). After stratification by the magnetic field intensity of MRI, subgroup analysis of the left OB volume revealed result similar to those from the subgroup analysis of the right OB volume, as distinct results were observed between 3.0 T MRI analysis (WMD = -17.648; 95% CI -27.896, -7.399; P_*Q*_ = 0.012; *I*^*2*^ = 84.3%) and 1.5 T MRI analysis (WMD = -4.566; 95% CI -10.234, 1.103; P_*Q*_ = 0.310; *I*^*2*^ = 16.3%; Fig 4D). Sensitivity analyses showed no significant changes in the pooled WMD or 95% CI upon the exclusion of any study. This result indicated that the overall pooled estimates were both stable and robust.

### OB volume in PD patients

The fixed-effects model was adopted because low heterogeneity was detected (P_*Q*_ = 0.974; *I*^*2*^ = 0%). The right OB volume was significantly larger than the left OB volume according to the pooled analysis (WMD = 1.618; 95% CI 0.264, 2.971; Fig 2C). The results of Begg’s test (P = 0.452), Egger’s test (P = 0.653), and funnel plots provided no evidence of publication bias (Fig 3C).

### OB volume in healthy controls

The OB volume in healthy controls was evaluated, and the pooled estimates showed no significant difference between the left and the right OB volume (WMD = 0.317; 95% CI -1.351, 1.986; Fig 2D) based on a fixed-effects model, which was applied because low heterogeneity was found (P_*Q*_ = 0.994; *I*^*2*^ = 0%). No significant publication bias was found according to Begg’s test (P = 0.707), Egger’s test (P = 0.595), or funnel plots (Fig 3D).

## Discussion

The OBs are paired, and their oval shape occupies the most anterior portion of the skull base, which receives inputs from sensory neurons that are located in the olfactory epithelium. The OB responds to odors and plays a central role in the processing of olfactory information [33]. Due to the constant development of MRI technology, MRI-based volumetric analyses serve as an accurate method to precisely measure OB volume, which correlates with olfactory function in humans [34]. The present comprehensive systematic review and meta-analysis assessed the difference in OB volume between PD patients and healthy controls.

This study demonstrated that based on the currently available evidence, both the right and the left OB volume were significantly smaller in PD patients than in healthy controls. The potential cause of this result would be a top–down mechanism, such that central nervous system processes may affect the OB volume. PD is traditionally considered as a progressive neurodegenerative disease that affects the motor system, with characteristic lesions in the substantia nigra pars compacta (SNpc). However, postmortem analyses suggested that PD is more than a movement disorder that progresses in a predictable sequence, for example, as described in the staging criteria of Braak et al. [8,35,36]. In this work, it was hypothetically predicted that PD initiates in the dorsal motor nucleus of the vagus nerve, as well as the anterior olfactory nucleus and OB, followed by the dispersal of dysfunction throughout the brainstem nuclei, ultimately reaching the SNpc. Moreover, Braak et al. [8,36,37] and Hawkes et al. [7] found that the initial PD-related changes simultaneously begin in the dorsal nucleus of the vagus nerve and in the olfactory nucleus and OB, where Lewy bodies are found. The aforementioned neurodegenerative process may contribute to decreases in olfactory structure volume and dysfunction of the olfactory system, and these events may precede motor syndromes in PD patients. The guidelines for the diagnosis and treatment of PD emphasize that early diagnosis and intervention prior to neurodegeneration in the SNpc may enable the halting, or at least delaying, of disease progression [38,39].

However, the results of this systematic review and meta-analysis should be interpreted prudently due to significant heterogeneity between studies. The *I*^*2*^-values for the comparisons of the right and the left OB volume between PD patients and healthy controls were 75.9% and 76.1%, respectively; these results suggested high between-study heterogeneity. To explore the potential sources of heterogeneity and to conduct a more precise analysis, subgroup analyses according to the country of origin of the participants and the magnetic field intensity of MRI were performed. In the subgroup analysis that according to country of origin, the right and left OB volumes remained consistent with the overall pooled estimate in the Chinese and German population but not in the Turkish population. It must be acknowledged that the between-study heterogeneity was effectively removed when the differences between these subgroups were considered, resulting in a modest *I*^*2*^ statistic and a P value >0.1. This finding suggested that the differences in the characteristics and the geographic origin of the participant might play a crucial role in the OB volume. Alternatively, this difference in OB volume between participants in distinct geographic regions may be caused by various factors, such as environmental and genetic factors, smoking behavior, lifestyle, or economic status, or by differences in the adjustment for other known or suspected risk factors between studies. When stratified according to the magnetic field intensity of MRI, the left and right OB volumes measured using a 3.0 T MRI, but not a 1.5 T MRI, were concordant with the overall results. The heterogeneity was decreased in the 1.5 T group but remained unchanged in the 3.0T group. Therefore, the magnetic field intensity of MRI, as a methodological factor, may not be a statistically significant source of heterogeneity. This finding was in stark contrast to the unmeasured or residual confounds of concern. In addition, the right OB volume was remarkably larger than the left OB volume, and this result indicated lateralized differences in the OB volume in PD patients. This finding is in line with evidence demonstrating that the right hemisphere is more important for higher-order processing of smell sensation than the left [40–42]. Furthermore, there was no significant difference in the lateralized OB volume in the healthy controls, and this result confirmed the findings in a previous study by Hummel et al. [43], who reported lateralized differences in olfactory function in approximately 20% of the general population.

The present systematic review and meta-analysis exhibit several important strengths. First, to the best of our knowledge, this research is the first to comprehensively evaluate whether there are significant differences in OB volume between PD patients and healthy controls. Second, the search strategy was exhaustive and was reproducible in multiple databases. Third, two independent reviewers performed study selection, study review, and data extraction to decrease potential biases and errors. Fourth, the quality of each study included was evaluated using the NOS, and all studies were found to be of high quality. Moreover, sensitivity analyses of the included studies showed no significant changes in the pooled estimates or 95% CIs upon excluding any of the studies, and this finding indicated that the overall results were both stable and robust.

However, this review should be interpreted within the context of its several limitations. First, this work was based on case-control studies, which was prone to be confounded by multiple factors, as reflected by the significant heterogeneity between studies. To explore the potential sources of heterogeneity, subgroup and sensitivity analyses were conducted. Nevertheless, residual confounding factors across studies inevitably remain a cause for concern in this study. Thus, between-study heterogeneity remains a formidable problem that may have affected the precision of the overall results. Second, although the search strategy was exhaustive and reproducible, it must be acknowledged that other relevant studies in the grey literature or unpublished articles may have been overlooked, and studies with negative findings may remain unpublished; these factors cannot be excluded from consideration. Third, the use of relatively few studies to perform subgroup analyses to explore potential confounders might have decreased the power for detecting sources of heterogeneity. Fourth, although the funnel plots and Egger’s and Begg’s tests provided no evidence of publication bias, it was very likely that the results were inherently weak due to the limited number of studies.

## Conclusion

Despite these limitations, this systematic review and meta-analysis provide evidence that both the right and the left OB volume in PD patients were significantly smaller than those in healthy controls. This conclusion is based on limited methodological quality and relatively few studies and is not definitive. Additional well-designed and well-conducted observational studies can further our understanding of the differences in OB volume between PD patients and healthy controls, and such an understanding will provide insight into the critical effects of the early identification and treatment of PD.

## Supporting Information

S1 Checklist PRISMA checklist. (DOCX)

S1 Appendix Search strategies. (DOCX)
