# Supplementary material for: Changes in Olfactory Bulb Volume in Parkinson’s Disease: A Systematic Review and Meta-Analysis
Source: PLoS One. 2016 Feb 22;11(2):e0149286. doi: 10.1371/journal.pone.0149286 (PMC4762676; doi:10.1371/journal.pone.0149286)
Supplement: S1 Appendix — (DOCX) [file pone.0149286.s002.docx]

**Appendix1.Search Strategies**

**PubMed**

1. "Parkinson Disease"[Mesh]
2. Parkinson Disease[Title/Abstract]
3. Parkinson's Disease[Title/Abstract]
4. Parkinsons Disease[Title/Abstract]
5. Idiopathic Parkinson Disease[Title/Abstract]
6. Idiopathic Parkinson's Disease[Title/Abstract]
7. Idiopathic Parkinsons Disease[Title/Abstract]
8. (Parkinson's Disease[Title/Abstract]) AND Idiopathic[Title/Abstract]
9. Parkinsonism[Title/Abstract]
10. Primary Parkinsonism[Title/Abstract]
11. (Parkinsonism[Title/Abstract]) AND Primary[Title/Abstract]
12. Lewy Body Parkinson Disease[Title/Abstract]
13. Lewy Body Parkinson's Disease[Title/Abstract]
14. Lewy Body Parkinsons Disease[Title/Abstract]
15. (Parkinson's Disease[Title/Abstract]) AND Lewy Body[Title/Abstract]
16. Paralysis Agitans[Title/Abstract]
17. PD[Title/Abstract]
18. parkinson*[Title/Abstract]
19. 1 OR 2 OR 3 OR 4 OR 5 OR 6 OR 7 OR 8 OR 9 OR 10 OR 11 OR 12 OR 13 OR 14 OR 15 OR 16 OR 17 OR 18
20. "Olfactory Bulb"[Mesh]
21. Olfactory Bulb[Title/Abstract]
22. Olfactory Bulbs[Title/Abstract]
23. (Bulb[Title/Abstract]) AND olfactory[Title/Abstract]
24. (Bulbs[Title/Abstract]) AND olfactory[Title/Abstract]
25. Main Olfactory Bulb[Title/Abstract]
26. Main Olfactory Bulbs[Title/Abstract]
27. (Bulb[Title/Abstract]) AND Main Olfactory[Title/Abstract]
28. (Bulbs[Title/Abstract]) AND Main Olfactory[Title/Abstract]
29. (Olfactory Bulb[Title/Abstract]) AND main[Title/Abstract]
30. (Olfactory Bulbs[Title/Abstract]) AND main[Title/Abstract]
31. Bulbus Olfactorius[Title/Abstract]
32. (Olfactorius[Title/Abstract]) AND Bulbus[Title/Abstract]
33. 20 OR 21 OR 22 OR 23 OR 24 OR 25 OR 26 OR 27 OR 28 OR 29 OR 30 OR 31OR 32
34. 33 AND volume[Title/Abstract]
35. 19 AND 34

**Embase**

1. 'parkinson disease'/exp
2. 'parkinson disease':ab,ti
3. 'parkinsons disease':ab,ti
4. 'idiopathic parkinson disease':ab,ti
5. 'idiopathic parkinsons disease':ab,ti
6. 'idiopathic':ab,ti AND 'parkinsons disease':ab,ti
7. 'idiopathic':ab,ti AND 'parkinson disease':ab,ti
8. 'primary parkinsonism':ab,ti
9. 'primary':ab,ti AND 'parkinsonism':ab,ti
10. 'lewy body parkinson disease':ab,ti
11. 'lewy body parkinsons disease':ab,ti
12. 'lewy body':ab,ti AND 'parkinsons disease':ab,ti
13. 'lewy body':ab,ti AND 'parkinson disease':ab,ti
14. 'paralysis agitans':ab,ti
15. PD:ab,ti
16. parkinson*:ab,ti
17. 1 OR 2 OR 3 OR 4 OR 5 OR 6 OR 7 OR 8 OR 9 OR 10 OR 11 OR 12 OR 13 OR 14 OR 15 OR 16
18. 'olfactory bulb'/exp
19. 'olfactory bulb':ab,ti
20. 'olfactory bulbs':ab,ti
21. bulb:ab,ti AND olfactory:ab,ti
22. bulbs:ab,ti AND olfactory:ab,ti
23. 'main olfactory bulb':ab,ti
24. 'main olfactory bulbs':ab,ti
25. 'main olfactory':ab,ti AND bulb:ab,ti
26. 'main olfactory':ab,ti AND bulbs:ab,ti
27. 'olfactory bulb':ab,ti AND main:ab,ti
28. 'olfactory bulbs':ab,ti AND main:ab,ti
29. 'bulbus olfactorius':ab,ti
30. olfactorius:ab,ti AND bulbus:ab,ti
31. 18 OR 19 OR 20 OR 21 OR 22 OR 23 OR 24 OR 25 OR 26 OR 27 OR 28 OR 29 OR 30
32. 31 AND volume:ab,ti
33. 17 AND 32
